# Standardizing registry data to the OMOP Common Data Model: experience from three pulmonary hypertension databases

**DOI:** 10.1186/s12874-021-01434-3

**Published:** 2021-11-02

**Authors:** Patricia Biedermann, Rose Ong, Alexander Davydov, Alexandra Orlova, Philip Solovyev, Hong Sun, Graham Wetherill, Monika Brand, Eva-Maria Didden

**Affiliations:** 1 Actelion Pharmaceuticals Ltd, Gewerbestrasse 16, CH-4123 Allschwil, Switzerland; 2 Odysseus Data Services, Inc., Cambridge, MA USA; 3 Janssen-Cilag Ltd, High Wycombe, UK

**Keywords:** Pulmonary hypertension, Registry, Observational data, Common data model, Data mapping

## Abstract

**Background:**

The Observational Medical Outcomes Partnership (OMOP) Common Data Model (CDM) can be used to transform observational health data to a common format. CDM transformation allows for analysis across disparate databases for the generation of new, real-word evidence, which is especially important in rare disease where data are limited. Pulmonary hypertension (PH) is a progressive, life-threatening disease, with rare subgroups such as pulmonary arterial hypertension (PAH), for which generating real-world evidence is challenging. Our objective is to document the process and outcomes of transforming registry data in PH to the OMOP CDM, and highlight challenges and our potential solutions.

**Methods:**

Three observational studies were transformed from the Clinical Data Interchange Standards Consortium study data tabulation model (SDTM) to OMOP CDM format. OPUS was a prospective, multi-centre registry (2014–2020) and OrPHeUS was a retrospective, multi-centre chart review (2013–2017); both enrolled patients newly treated with macitentan in the US. EXPOSURE is a prospective, multi-centre cohort study (2017–ongoing) of patients newly treated with selexipag or any PAH-specific therapy in Europe and Canada. OMOP CDM version 5.3.1 with recent OMOP CDM vocabulary was used. Imputation rules were defined and applied for missing dates to avoid exclusion of data. Custom target concepts were introduced when existing concepts did not provide sufficient granularity.

**Results:**

Of the 6622 patients in the three registry studies, records were mapped for 6457. Custom target concepts were introduced for PAH subgroups (by combining SNOMED concepts or creating custom concepts) and World Health Organization functional class. Per the OMOP CDM convention, records about the absence of an event, or the lack of information, were not mapped. Excluding these non-event records, 4% (OPUS), 2% (OrPHeUS) and 1% (EXPOSURE) of records were not mapped.

**Conclusions:**

SDTM data from three registries were transformed to the OMOP CDM with limited exclusion of data and deviation from the SDTM database content. Future researchers can apply our strategy and methods in different disease areas, with tailoring as necessary. Mapping registry data to the OMOP CDM facilitates more efficient collaborations between researchers and establishment of federated data networks, which is an unmet need in rare diseases.

**Supplementary Information:**

The online version contains supplementary material available at 10.1186/s12874-021-01434-3.

## Background

Evidence generated from observational, real-world data can be highly insightful and is increasing in importance, particularly in rare diseases where information is limited [1–3]. The analyses of observational data, such as administrative claims databases, electronic healthcare records, or registries, offer the potential for patient and disease characterization, drug surveillance, and comparison of the effectiveness or safety of interventions [4–7]. The gathering and analyses of real-world data to provide real-world evidence has been identified as a major priority in the twenty-first Century Cures Act of 2016 [8, 9] and in the concept paper for the proposed Cures Act 2.0 [10] in the US, with the aim of accelerating drug development and innovation. The analyses of data from multiple healthcare data sources can be an efficient and cost-effective approach for evidence generation [11]; however, it is difficult to combine data without losing information because each database has its own original purpose, objectives, structure, and terminology. A logical solution to address this problem would be to store data in a standardized format, such as a common data model (CDM). A CDM is an informational model that allows transformation of data contained in different databases to a common format, in which all coding and vocabulary are pre-specified and standardized [12], and can be applied to all data irrespective of product or therapy area. Transforming data sources into CDM is a convenient way to allow analyses across multiple sources.

Several data networks have been established with a view to improving the standardization of observational healthcare data and to establish shared open-source tools that facilitate collaborative advancement of disease understanding and research. Examples include the Sentinel initiative of the US Food and Drug Administration (FDA) and the European Union Adverse Drug Reactions (EU-ADR), which were both developed to monitor medical products [13–15], as well as the European Network of Centres for Pharmacoepidemiology and Pharmacovigilance (EnCePP), the National Patient-Centred Clinical Research Network (PCORnet) and the Health Care Systems Research Network (HCSRN; formerly HMO Research Network) [16–18]. Similarly, the Observational Medical Outcomes Partnership (OMOP), a public-private partnership between the FDA, academic institutions, database owners, and pharmaceutical companies, and the subsequent Observational Health Data Sciences Informatics (OHDSI) collaborative aimed to utilize existing observational healthcare data for safety surveillance [19–22]. Most of the above-mentioned networks have developed their own CDM to meet their aims.

Studies comparing different CDMs showed that the OMOP CDM met most of the desired criteria (including content coverage, integrity, flexibility, ease of querying, standards compatibility, and ease/extent of implementations, privacy and linkage) for data sharing and across use cases [23, 24]. All other investigated CDMs (FDA Mini-Sentinel/Sentinel, PCORnet, and the Clinical Data Interchange Standards Consortium study data tabulation model (SDTM) – which is the established standardized format and organization of clinical trial data [25]) scored lower for observational healthcare data than the OMOP CDM for content coverage, cost or clinical outcome measures, data linkage and/or case definition [23, 24]. Advantages of standardizing to the OMOP CDM include that users do not need to understand all database-specific schema details, firmly controlled terminology exists making datasets within the OMOP CDM comparable, and the concepts are freely available for researchers to access. Furthermore, in a replication analysis it has been reported that up to 80% less programming time was required in OMOP CDM than in the raw data [26].

Pulmonary hypertension (PH) is a progressive and ultimately fatal disease, which is classified into five clinically diverse groups, including the rare subgroups pulmonary arterial hypertension (PAH) and chronic thromboembolic pulmonary hypertension (CTEPH) [27]. In PH, there are a number of recently completed and currently ongoing registries, which are formatted to the SDTM. Registries are vital sources of information that can provide insight into the characteristics and longitudinal trends of a specific patient population [28, 29]. The OPsumit® USers (OPUS) registry (NCT02126943) was a prospective drug registry initiated in response to an FDA post-marketing requirement to characterize the safety profile of the endothelin receptor antagonist, macitentan, in a real-world setting. The OPsumit® Historical USers (OrPHeUS) cohort study (NCT03197688) was a retrospective medical chart review designed to supplement OPUS with data from additional patients to achieve the necessary sample size for the primary outcome. EXPOSURE is an ongoing, international, multicentre, prospective, real-world, observational study (EUPAS19085) in patients with PAH following marketing authorization of the IP-receptor agonist, selexipag, by the European Medicines Agency (EMA). To date, predominantly large administrative claims or electronic medical records databases have been converted to a CDM [6, 7, 26, 30, 31], while the mapping of registries has been seldom undertaken and presents additional challenges compared with coded databases. One study has mapped the German PH registry to the OMOP CDM from source data in Microsoft Access with limited details [32] and a second study has described the harmonization of data from the US-based SEARCH diabetes registry and the observational, multi-centre youth diabetes registry of patients in India to the OMOP CDM; however, the standardization process itself was not described [33].

The SDTM is the standard format used for clinical trial data and while it defines a standard structure for its tables and its framework allows flexibility, this structure is achieved through the wider availability of tables and columns following suggested patterns and these are tailored to clinical trial data. In contrast, the core structure of the OMOP CDM is fixed and the number of tables, and their respective fields, are finite, which allows for easier and consistent application development due to the underlying rigidity of the data model. Moreover, the OMOP CDM was designed for observational data (including claims and electronic healthcare records), and a number of observational healthcare databases have already been mapped to OMOP CDM, including a PH registry [30–32, 34–36]. In addition, a study comparing the utility of various CDMs for the purposes of comparative effectiveness research found the OMOP CDM to be the best-suited [37]. Importantly, in recent years, there has been evolution towards federated networks that foster research using disparate databases and are therefore in need of standardised data assets. Federated data networks, such as the European Health Data Evidence Network (EHDEN) [38], are considered as the future for research collaboration and real-world evidence generation in rare diseases. Therefore, the mapping of PH registry databases to the OMOP CDM allows researchers to use a wider base to generate more robust conclusions, as they can perform analyses across the many disparate real-world data assets available in OMOP CDM format. This can represent a particular advantage in a disease like PH, as it is comprised of rare and diverse subgroups [1, 27]. The data available on PH subgroups are limited; databases of patients with PH and its subgroups are often small and from disparate regions, without the possibility of readily pooling the data. We aim to describe our experience in transforming data from the OPUS, OrPHeUS and EXPOSURE observational studies from the SDTM to the OMOP CDM, and to highlight the benefits and challenges to this novel process.

## Methods

### Data sources

Three registries, formatted to the SDTM, were converted to the OMOP CDM, and are summarized in Table 1. For all datasets, the raw data are available in SDTM format before patients are excluded based on eligibility criteria. The SDTM format is determined by the design of the study and case report form (CRF), for example, tick boxes or free text and mandatory or optional fields. Therefore, all events are captured in SDTM, including those performed (e.g., ‘yes’ selected, or box ticked) and not performed (e.g., ‘no’ selected, or box left unticked), since it is of interest to these studies whether an assessment was performed. The flexibility of free text and optional fields means that there are sometimes missing or partially missing data in the SDTM.

**Table 1:** Overview of the OPUS, OrPHeUS and EXPOSURE databases

	OPUS	OrPHeUS	EXPOSURE
Study identifier	NCT02126943	NCT03197688	EUPAS19085
Study type	Multi-centre, prospective drug registry of PH patients newly treated with macitentan	Multi-centre, retrospective medical chart review of PH patients newly treated with macitentan	Multi-centre, prospective cohort study of patients with PAH newly treated with either selexipag or any other PAH-specific therapy
Region	USA	USA	Europe and Canada
Date ranges	Apr 2014 – Apr 2020	Oct 2013 to Mar 2017	Sept 2017 – ongoing^a^
Patients in database, N	2722	3142	758
Age at enrolment in database, mean (SD), years [N]	60.93 (14.15) [*N* = 2682]	60.21 (15.06) [*N* = 3060]	59.96 (15.07) [*N* = 752]
Gender, %			
Female	71.6	72.2	68.6
Male	26.9	25.2	31.3
Missing	1.4	2.5	0.1
Source data format			
Conditions	MedDRA and free text	MedDRA and free text	MedDRA and free text
Drugs	WHODrug and free text	WHODrug and free text	WHODrug and free text

^a^Data cut-off for this analysis was 30 Nov 2019

Percentages might not add up to 100.0% due to rounding

*MedDRA* Medical Dictionary for Regulatory Activities; *OPUS* OPsumit® USers *OrPHeUS* OPsumit® Historical USers; *PH* pulmonary hypertension; *PAH* pulmonary arterial hypertension; *SD* standard deviation; *USA* United States of America; *WHODrug* World Health Organization Drug Dictionary

The OPUS, OrPHeUS and EXPOSURE registries were reviewed and approved by the relevant ethics committee/Institutional Review Boards for each respective database. Patients in OPUS and EXPOSURE provided informed consent for their protected, anonymized health information to be stored in a computer database and analysed by researchers and healthcare professionals, and for the results of these analyses to be published. The requirement for informed consent was waived for OrPHeUS to enable inclusion of patients who had died in this retrospective observational study.

### OMOP CDM

**Fig. 1:**
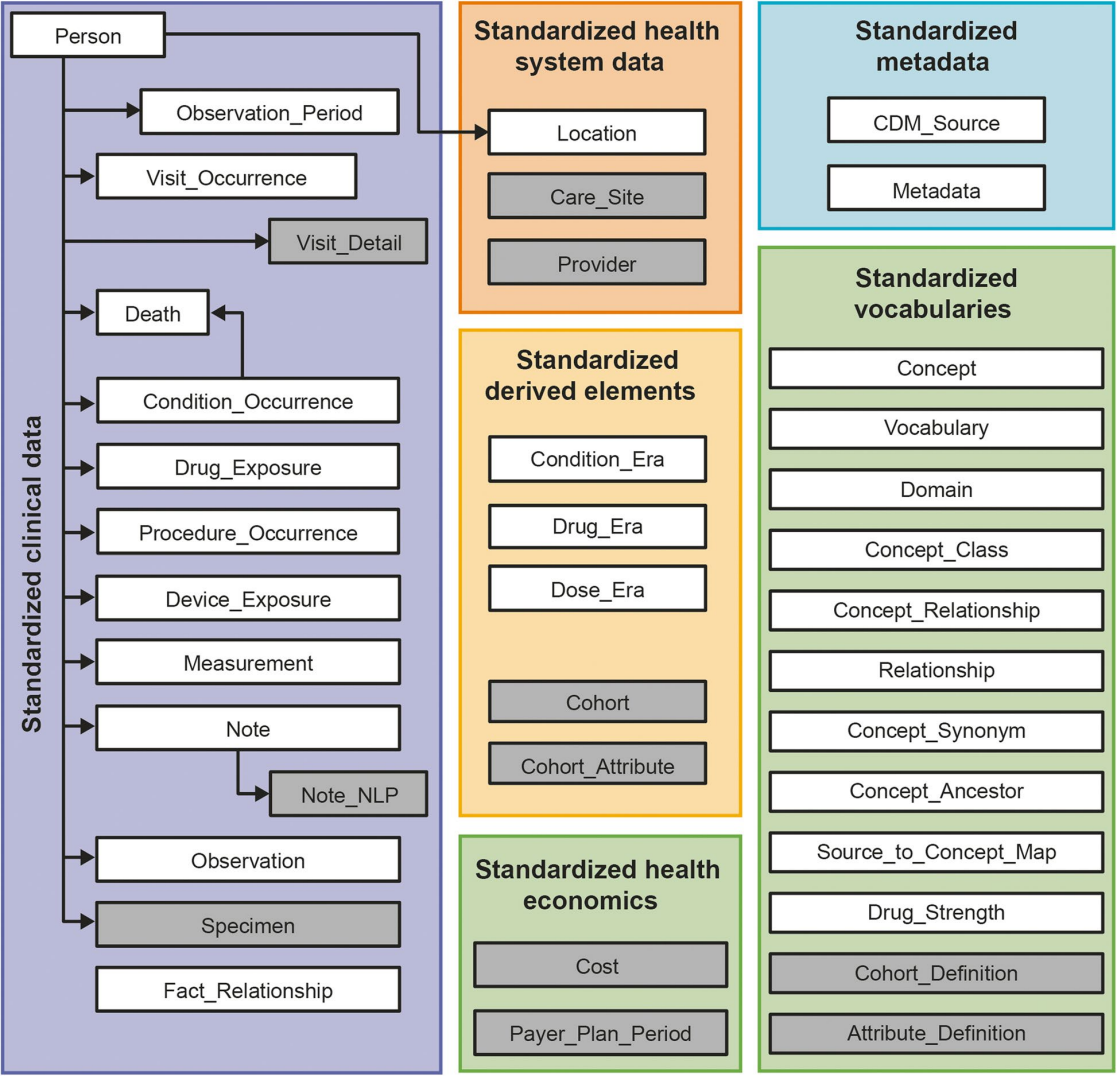
Standardized tables and vocabularies available in the OMOP CDM. Tables and vocabularies in grey were not populated/used during this analysis. Table adapted from The Book of OHDSI, chapter 4 [39]. OMOP CDM, Observational Medical Outcomes Partnership Common Data Model

The OMOP CDM is a patient-centric model, meaning that every clinical event has, at a minimum, a patient identifier (ID number guaranteeing anonymity) and a date. This also allows healthcare events to be viewed over a given time horizon for each individual [22, 37]. Table 2 defines the key terms used in OMOP CDM. Patient and medical information are organized into ‘domains’ in the OMOP CDM, which are stored in domain-specific tables and fields, and examples include a drug domain or condition domain. In turn, these domain-specific tables are populated with ‘standard concepts’, which have a unique domain assignment dictating the table that it is recorded in [22, 37]. In the OMOP CDM, the content of each patient record is transformed to the machine-readable format so that they are represented as ‘concepts’, which are stored in CONCEPT table [22, 37]. The tables within the OMOP CDM contain equivalent information recorded in multiple ways at once: as a ‘source value,’ a ‘source concept,’ and as a ‘standard concept’ (Table 2) [22, 37]. The OMOP standardized vocabulary is a common repository of all OHDSI-supported vocabularies and ensures standardization (Table 2) [22, 37]. The standard tables contained in the OMOP CDM are shown in Fig. 1.

**Table 2:** Definitions of OMOP CDM terminology

	Definition
Standardized vocabulary	A common repository of all terminologies used within the OMOP CDM consolidated into a common format
Concept	A term defined in a medical terminology
Source values	The verbatim representation of an event record in the source data using the original codes from public code systems (e.g. ICD, National Drug Code, Current Procedural Terminology 4th edition) or locally controlled vocabularies that are not used in analysis and only provided for convenience and quality purposes
Source concepts	Source concepts represent the terms in the common healthcare terminology systems that were used in the source database, and are often found in the OMOP vocabularies as non-standard concepts
Standard concepts	Standard concepts are the respective target concepts that define the unique meaning of a clinical entity and are typically drawn from existing public terminologies such as SNOMED or created within OMOP as extension concepts if no suitable target is available
Custom concepts	Clinical terms that are custom generated for information that is not represented in the standardized vocabulary
Classification concepts	These are non-standard concepts that do not represent the data in the OMOP CDM, but instead are part of the standard concepts hierarchy, and can be used for hierarchical queries to search for a certain concept. They can be used for analysis, but they have no full equivalent ‘maps to’ links to standard concepts. Hence, classification concepts, such as MedDRA terms, require a degree of manual mapping

*ICD* International Classification of Disease; *MedDRA* Medical Dictionary for Regulatory Activities; *OMOP CDM* Observational Medical Outcomes Partnership common data model; *SNOMED* Systematized Nomenclature of Medicine Clinical Terms

### Sample selection

In order to map the data in the registries to the OMOP CDM, patients with important data completely missing (such as birth year, or treatment start and end dates) were excluded as well as those who violated inclusion/exclusion criteria of the original registry (but were mistakenly enrolled before being excluded from further data entry, and thus had partial data in the SDTM database). These patients were not part of the target population for the registry. Duplicate records for the same patient within a registry were also excluded. For the OrPHeUS and OPUS finalized studies, mapping to OMOP CDM was performed initially in September 2019 and with the final data mapped in October 2020. The EXPOSURE database was first mapped in August 2019, with a refresher in December 2019. An annual data refresh is planned for EXPOSURE, which is an ongoing study.

### Mapping methods

The tools and programs used for the mapping process are summarized in the Supplementary Appendix. The following roles were required for expertise in the different parts of the mapping process: project manager; epidemiology data analyst; biostatistician, developer; tester; medical terminologist; medical expert; source data expert, and observational data scientist.

**Fig. 2:**
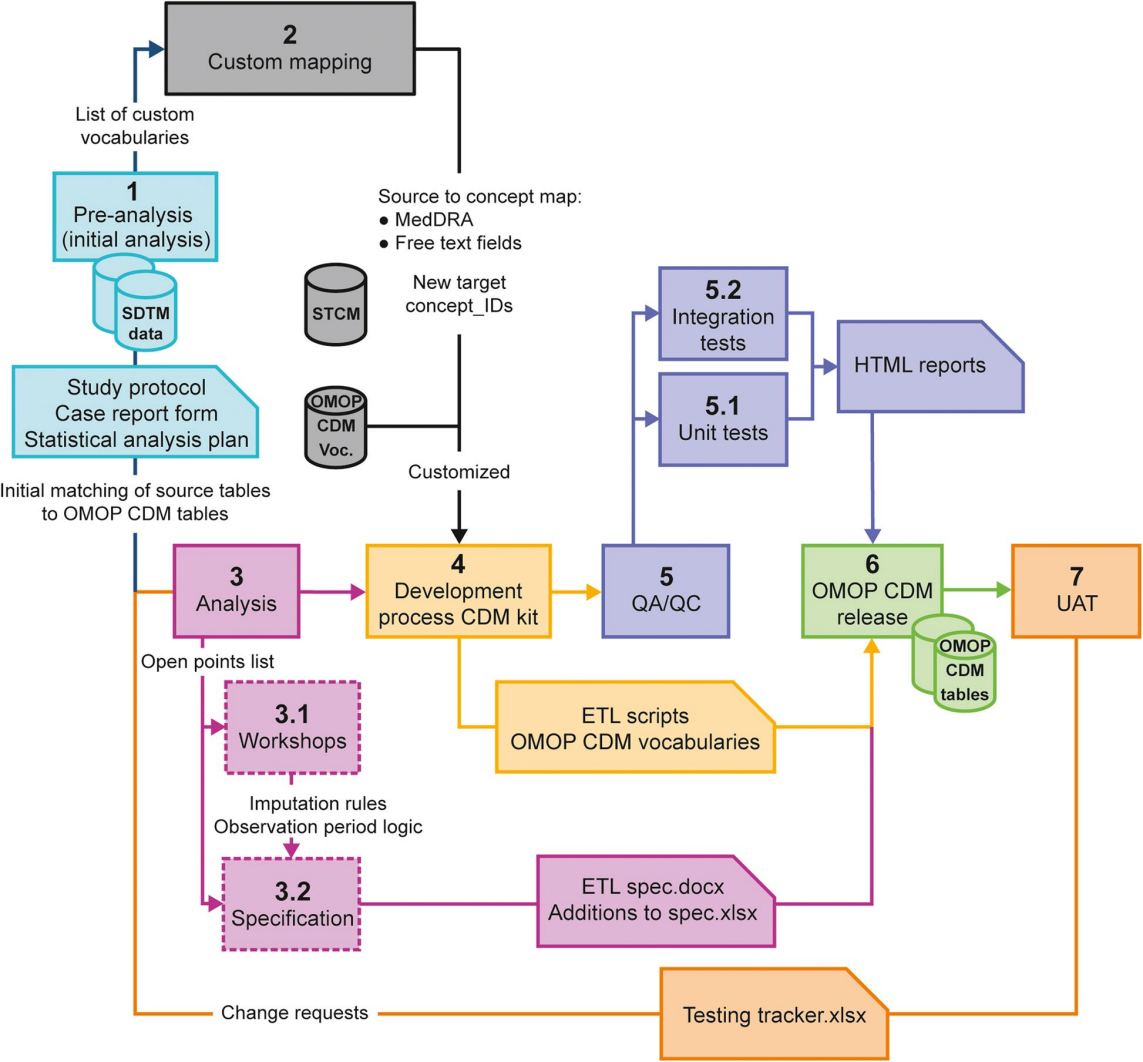
An overview of the process of mapping registry data to the OMOP CDM. CRF, case report form; ETL, Extract, Transform, Load; HTML, Hypertext Markup Language; OMOP CDM; Observational Medical Outcomes Partnership Common Data Model; QA, quality assessment; QC, quality control; SAP, statistical analysis plan; SDTM, study data tabulation model; UAT, user acceptance testing

**Fig. 3:**
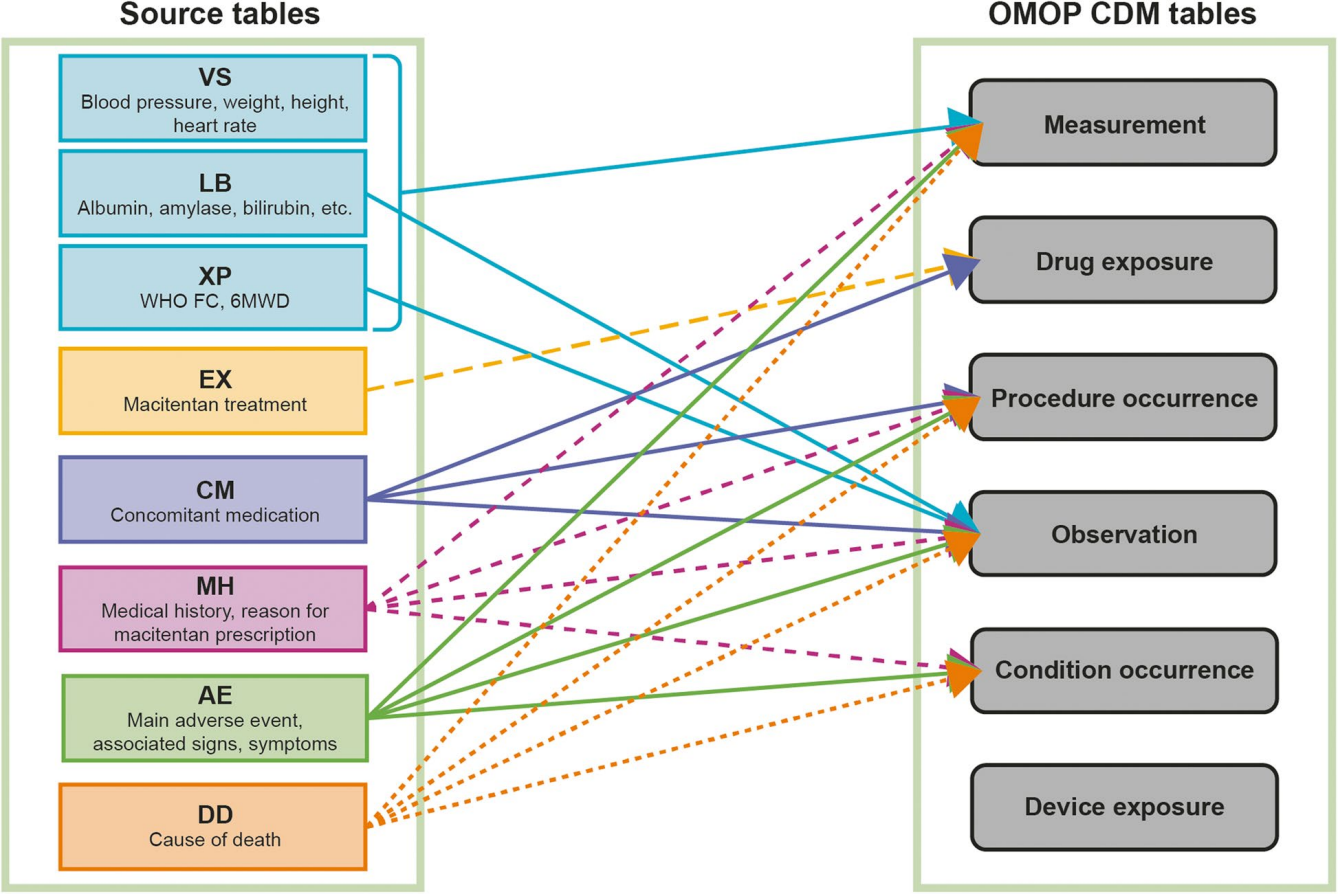
Example mapping from the OPUS SDTM-format database to the OMOP CDM. For instances where source tables have multiple options for OMOP CDM tables, a decision on which OMOP CDM table to store the record in is decided on a case-by-case basis using OMOP CDM conventions, if available, or customization. AE, adverse event; CM, concomitant medication; DD, death details; EX, exposure to study medication; LB, laboratory data; MH, medical history; OMOP CDM, Observational Medical Outcomes Partnership Common Data Model; VS, vital signs; XP, pulmonary arterial hypertension

An overview of the 7-phase process of mapping registry data in the SDTM format to the OMOP CDM is shown in Fig. 2 and further details are in the Supplementary Appendix. In the first, pre-analysis phase, source documentation and the SDTM were reviewed and a list of questions to discuss with the source data experts was prepared. From this, initial matching of source tables to OMOP CDM tables was performed and a list of custom vocabularies and sets of values to be custom mapped by medical experts was determined. The custom mapping of source values was performed when source concepts and/or codes were not available with equivalent standard concepts in the standardized OMOP CDM vocabularies. A specific example of mapping source tables to OMOP CDM tables is shown in Fig. 3. Currently, all-level MedDRA terms, which are used in the OPUS, OrPHeUS and EXPOSURE databases, are not an OHDSI-supported vocabulary but are considered ‘classification concepts’ (Table 2) in OMOP. As classification concepts, MedDRA terms have no full equivalent ‘maps to’ links to standard concepts, meaning that the MedDRA codes have no direct translation to OMOP standardized vocabulary for conditions and laboratory data (SNOMED, LOINC) and, thus, a degree of manual mapping was required. This process is described in further detail in the results.

As the OMOP CDM requires complete dates, imputation rules were developed where this information was missing. Complete or partial (only year or year and month available in original record) missing dates for adverse events, laboratory tests, medical history, medications, hospitalizations, assessments and procedures were imputed. In addition, custom concepts were generated to capture information such as adverse event severity and causality, for which the OMOP CDM lacks a way to capture concisely.

Data refreshes (e.g., for EXPOSURE) involve similar steps to the original mapping. The version of the OMOP vocabulary is updated, if required, and the source data are closely examined for any significant changes compared with the initial data set. Unknown source codes are extracted, as well as any codes that have changed their description, and mapping is provided for these codes. The mapping is aligned to the current conventions and ETL specifications, and the custom concepts, CONCEPT_RELATIONSHIP and CONCEPT_ANCESTOR tables are rebuilt. Finally, the ETL code is adjusted and run on the new data.

### Quality assessment methods

Descriptive summary statistics were obtained for the SDTM and OMOP CDM data sets, programmed in R (version 3.4.3), to compare consistency between source data and mapped data.

## Results

### Database-specific information

Of the 2722, 3142, and 758 patients in OPUS, OrPHeUS, and EXPOSURE, respectively, a total of 132 were excluded for violating original study inclusion/exclusion criteria and 33 were excluded due to OMOP CDM mapping conventions (Table 3). To note, the number of patients who were excluded due to violation of the original study inclusion/exclusion criteria was lower for EXPOSURE as all but one patient who were mistakenly enrolled in EXPOSURE were deleted from the electronic data capture prior to transfer of data to the SDTM database. The total number of mapped patients was 6457 (Table 3). The main challenges and solutions, described herein, are summarized in Table 4.

**Table 3:** Data exclusion in the mapping process to the OMOP CDM

	OPUS	OrPHeUS	EXPOSURE
Patients in database, N	2722	3142	758
Patients violating original study inclusion criteria, n (%)	46 (1.7)	85 (2.7)	1 (0.1)
Patients excluded during mapping to OMOP CDM, n (%)	2 (0.1)	25 (0.8)	6 (0.8)
Total patients mapped, n (%)	2674 (98.2)	3032 (96.5)	751 (99.1)

*OMOP CDM* Observational Medical Outcomes Partnership common data model; *OPUS OPsumit® USers*; OrPHeUS, OPsumit® Historical USers

**Table 4:** Summary of the main challenges involved in mapping registry data to the OMOP CDM, and their solutions

Challenge	Solution
Mapping incomplete dates	Create standardized imputation rules to ‘restore’ missing dates
Mapping of MedDRA codes (classification concepts) to standard concepts	Automated mapping from the unified medical language system to the ICD-10, ICD-10-Clinical Modification or SNOMED vocabulary with crosslinks and name matching, followed by further expert review and additional manual mapping
Mapping of free text e.g. from medication tables	Free text extracted, custom mapped and contextualized via CONCEPT_RELATIONSHIP and CONCEPT_ANCESTOR tables
Mapping of non-existing OMOP vocabulary e.g. PH subgroups	Used either a combination of SNOMED concepts (e.g. PAH and underlying cause), or a new custom concept (e.g. drug- and toxin-induced PAH)
Mapping information that is related to another piece of information e.g. severity of an event	Separate clinical facts are stored in their appropriate domain and a link is added in the FACT_RELATIONSHIP table
Capturing the information that a procedure was not performed	This information was excluding from mapping, per the OMOP convention, but is planned for mapping in a future update

*ICD* International Classification of Disease; *MedDRA* Medical Dictionary for Regulatory Activities; *OMOP CDM* Observational Medical Outcomes Partnership common data model; *SNOMED* Systematized Nomenclature of Medicine Clinical Terms

### Applied imputation rules

Workshops were scheduled with the registry study team members to define imputations rules, with our highest priority being to keep imputation rules as consistent as possible across the three registries. Existing imputation rules were used if available and appropriate (e.g., from the statistical analysis plan of the individual registry), and new imputation rules were developed by considering the unique study design and the CRF structure. Imputation rules were mainly developed based on the following four strategies: i) extraction of dates from free text fields (in instances where the date and timestamp are stored in the database as a text value); ii) usage of time points available in the SDTM (e.g., ‘before patient discontinuation’ or ‘within 3 months of baseline’); iii) imputation based on the previous interval of taking a certain drug; iv) comparison of the year and/or month of a medical event with pre-defined reference time points such as date of death, drug initiation or end date, date of last available information or last follow-up visit, and end of study date.

For example, laboratory test dates were imputed as follows: if the SDTM time point for drug initiation was present and had, for example, the same month and year as the laboratory test date then the missing date was imputed with the drug initiation date. If the laboratory test time point was the last available information before study end then the missing date was imputed with either the drug end date or study end date, whichever occurred first. In addition, imputation of drug initiation dates was performed as follows: if the day and/or month was missing and the year was available, then the missing date was imputed with the first day of that month (for missing day only) or 1 January (for missing day and month) for the same year. However, if this date was before the end date of the previous drug interval, then the drug initiation date was imputed with the end date of the previous drug plus 1 day.

Furthermore, for patients with recorded death in the database, but with a partly or completely missing date of death, the date of death for patients was imputed based on several algorithms. Firstly, the date of death may have been available in the following sources: the study CRF, the drug safety database or as the date of an adverse event with a fatal outcome; if available, a partially missing date (day, month and/or year) was gathered from these sources. Secondly, if several possible dates from the same patient had the same number of missing parts (day and/or month), then the following hierarchy was applied: death details from the study CRF; adverse events with a fatal outcome from the study CRF; death details from the drug safety database. Finally, in cases where the date of death had still not been determined, it was imputed with the date of last available information. For partially missing dates where the imputed death date occurred prior to the date of last available information, then the imputed death date was replaced with the date of last available information.

### Mapping process and customization

Some of the registry data captured in SDTM format could not be fully accommodated in the OMOP CDM design, and had to be stored in the OMOP CDM without adjustment of additional tables or fields. Table 5 contains the most frequent cases and ways in which this information was transferred and stored in the OMOP CDM.

**Table 5:** Examples of the most frequent data transferred from the CDSIC SDTM to the OMOP CDM

Information transfer required	OMOP CDM solution used	Comments on solution
Additional characteristic of a medical event	• The characteristic of an event recorded as: *observation_concept_ID* • The assessment of medical event recorded as: *value_as_concept_ID* • Linking of the characteristic and the assessed medical event via *FACT_RELATIONSHIP*	The characteristic of an event could be its severity or seriousness or status (stable / unstable). Multiple characteristics can be mapped to allow all data to be captured. For example, a moderate-severity renal function impairment was mapped in the OMOP CDM as: CONDITION_OCCURRENCE: 4030518 - ‘*Renal impairment*’ OBSERVATION: *Observation_concept_ID* = 4022772 - ‘*Condition severity*’ *Value_as_concept_ID* = 4285732 - ‘*Moderate*’ FACT_RELATIONSHIP: 4030518 - ‘*Renal impairment*’; 4022772 - ‘*Condition severity*’
Registry enrolment date	• Registry enrolment fact recorded as: *observation_concept_ID* • Name of the study recorded as: *value_as_string*	The following concept_IDs and descriptions can be used: 44807982 - ‘*Participant in research study’* 4090379 - ‘*Patient entered into trial*’
Date of informed consent	• Informed consent fact recorded as: *observation_concept_ID* • Name of the study recorded as: *value_as_string*	*The following concept_IDs and descriptions can be used:* 44811375 - ‘*Consent given to participate in research study’* 4163733 - ‘*Patient consented to clinical trial’*
Reason for registry discontinuation	• Registry discontinuation fact recorded as: *observation_concept_ID* • Reason for discontinuation recorded as: *value_as_concept_ID*	The following concepts can be used: 44810922 - ‘*Participation in research study completed’* 44810920 - ‘*Withdrawn from research study*’ 40482840 - ‘*Completion of clinical trial’* 4087907 - ‘*Patient withdrawn from trial’*
Relationship to <entity> of medical event	• The relationship to an entity was recorded as: *observation_concept_ID* • The kind of relationship was recorded as: *value_as_concept_ID* • Linking of the kind of relationship and relation to an entity medical event via *FACT_RELATIONSHIP*	*<entity >* could include, for example, hospitalization or an adverse event A new custom concept ‘*Relationship to < entity >* ‘was created since there was no proper concept among existing ones. The *value_as_concept_ID* field could contain answers like: 45878245 - ‘*No*’ 45877241 - ‘*Unlikely*’ 45877994 - ‘*Yes*’ 4162850 - ‘*Possible*’ or Specific concepts denoted medical events like: 4322024 - ‘*Pulmonary hypertension’* 45885208 - ‘*Other reason’*
Outcome of medical event	• Transferred to the appropriate *EVENT* table • Linking of the outcome of medical event and the medical event via *FACT_RELATIONSHIP*	Outcomes can be stored as events in the OMOP CDM event table or specific *concept_ID*. 4231813 - *‘Adverse incident outcome categories*’ can be used in combination with the *value_as_concept_ID* field that stores the outcome itself.
Method of measurement	• The measurement recorded as: *measurement_concept_id* • The method of taking this measurement recorded as: *observation_concept_id* • Linking of the method of measurement and the assessed measurement via *FACT_RELATIONSHIP*	For example, a mPAP measurement derived from the thermodilution technique was mapped in the OMOP CDM as: MEASUREMENT to map XP measurement: *Measurement_concept_id =* 3028074 - ‘*Pulmonary artery Mean blood pressure”* OBSERVATION to map method: *Observation_concept_ID* = 4122989 - ‘*Thermodilution technique*’ FACT_RELATIONSHIP to link XP test to its method of measurement: *domain_concept_id_1* = 1147304 - '*Observation*' domain_concept_id_2* =* 1147330 - '*Measurement*' relationship_concept_id = 4152892 - ‘*Measurement method*’

*OMOP CDM* Observational Medical Outcomes Partnership Common Data Model; *XP* pulmonary arterial hypertension

To map MedDRA codes to standard concepts, mapping automation from the unified medical language system to the ICD-10/ICD-10-Clinical Modification or SNOMED vocabulary with crosslinks and name matching, with further expert review and additional manual mapping were performed. Concomitant and study medication data were encoded in the source data using the World Health Organization Drug Dictionary (WHODrug) vocabulary, which is not an OHDSI-supported vocabulary. In addition, the free text from medication tables as well as, for example, adverse events, laboratory tests, reasons for death, medical history, clinical events (that were not MedDRA coded) were required to be extracted and custom mapped, and contextualized via CONCEPT_RELATIONSHIP and CONCEPT_ANCESTOR tables if needed.

OMOP standardized vocabulary was used during the mapping process, and custom concepts were only generated when granular information could not be accurately mapped with existing vocabulary (such as for the different subgroups of PAH). In these cases, either a combination of SNOMED concepts was used for differentiation, e.g., PAH associated with connective tissue disease required PAH plus connective tissue disease overlap syndrome, or for PAH associated with congenital heart disease, PAH plus history of surgically corrected congenital heart defect was required. When mapping was not possible, custom concepts were introduced, such as for the disease subgroups of drug- and toxin-induced PAH and PH with unclear and/or multifactorial mechanisms (Group 5). Similarly, the standardized vocabulary did not contain an appropriate target concept for WHO functional class, which is used to assess the severity of PH, and custom concepts were introduced to address this.

All custom concepts were incorporated into the concept’s hierarchy, with custom CONCEPT_RELATIONSHIP and CONCEPT_ANCESTOR tables. This process allows users to easily identify relationships between variables, e.g., between conditions and subclasses of conditions, which facilitates the identification of specific patient cohorts for further analysis. The custom concept ‘drug- and toxin-induced PAH’, for instance, was integrated into the vocabulary as a descendent of the standardized standard concept ‘PAH.’ However, custom concepts operate only in the CDM instance, or group of instances, they were introduced to and cannot be used in the OMOP network studies. Once the OHDSI community identify the need for wide usage, these concepts can be integrated to the official OMOP vocabulary.

FACT_RELATIONSHIP tables were used to capture clinical information such as aetiology, and create links between treatment and an additional characteristic of an event, such as severity, reasons for dose change, hospitalization (causality), and the outcome of the adverse event. First, the separate clinical facts were stored in their appropriate domains, and second, the link between them was added in the FACT_RELATIONSHIP table.

With respect to the measurement and condition tables, the OMOP CDM does not differentiate between procedures or conditions that were not performed or not reported. The OMOP CDM convention is to only include events that have actually occurred; records about the absence of an event, or the lack of information, were, therefore, not mapped to the OMOP CDM.

### Quality assessments

The mapping process resulted in limited data exclusion, but a considerable consolidation of information (Table 3); most information was able to be supported by the OMOP CDM, either directly, or indirectly, via customization.

For condition codes, 10,659, 4013, and 449 unique source values (MedDRA codes or unique wording) were in the OPUS, OrPHeUS and EXPOSURE databases, respectively (Table 6). In the mapping process, if source values were the same, but appeared in different wording, they were mapped to the same concept_ID; accordingly, only 3698, 2704 and 337 unique concept_IDs were included in the OMOP CDM as a result of cross-linking, name matching, and custom mapping, for the OPUS, OrPHeUS and EXPOSURE records, respectively (Table 6). Therefore, 65% (OPUS), 33% (OrPHeUS) and 25% (EXPOSURE) of source values for condition codes were redundant and mapped to an existing concept_ID during this consolidation process. In total, 199,165 unique source records for condition codes were mapped to 108,657 unique OMOP CDM records (Table 6). Similarly, for drug codes, 51,612 unique source records were mapped to 46,360 unique OMOP CDM records (Table 6).

**Table 6:** Quality assessment results

**Condition codes**	**OPUS**	**OrPHeUS**	**EXPOSURE**
Unique source values in SDTM database, N	10,659	4013	449
Unique concept_IDs in OMOP CDM, n	3698	2704	337
Unique custom concept_IDs in OMOP CDM, n	6	6	5
Database-specific unique custom concept_IDs^a^, n	0	0	3
Unique source records in SDTM database, N	128,858	41,960	28,347
Unique OMOP CDM records, n	62,048	41,993	4616
Unique source records, n	55,789	41,608	4394
**Drug codes**	**OPUS**	**OrPHeUS**	**EXPOSURE**
Unique source values in SDTM database, N	2768	894	2496
Unique concept_IDs in OMOP CDM, n	1514	640	1050
Unique custom concept_IDs in OMOP CDM, n	4	4	0
Database-specific unique custom concept_IDs^a^, n	3	0	0
Unique source records in SDTM database, N	26,276	16,498	8838
Unique OMOP CDM records, n	23,368	15,169	7823
Unique source records, n	23,083	16,005	7537

^a^Unique custom concept_IDs that were only used on one database; OPUS and OrPHeUS used the same custom condition concept_IDs

The high percentage of records lost from the EXPOSURE database was due to the large amount of events in the clinical events table that were either marked as ‘unknown’ or ‘not occurred’ and were thus not incorporated, as per OMOP CDM convention

For OrPHeUS condition codes, the number of unique OMOP CDM records is higher than the number of unique source records in the SDTM database because (i) there are very few events that are marked as ‘unknown’ or ‘not occurred’, hence the vast majority of events are mapped to OMOP CDM and (ii) some of the source records can be mapped to more than one target concept_IDs. For example, source code MedDRA 10057688 - ‘Catheter site discharge’ is mapped to 4249456 - ‘Complication of catheter’ and 4183956 - ‘Skin discharge’

For example: source code MedDRA 10057688 - Catheter site discharge is mapped to 4249456 - Complication of catheter and 4183956 - Skin discharge

*OMOP CDM* Observational Medical Outcomes Partnership Common Data Model; *OPUS OPsumit® USers* OrPHeUS, OPsumit® Historical Users

The total percentage of excluded records from OPUS, OrPHeUS and EXPOSURE when mapping to the OMOP CDM was 35%, 7% and 52%, respectively (Supplementary Tables 1–3). The high percentage of records excluded from the OPUS and EXPOSURE databases was due to the large number of records in the clinical events table that were either marked as ‘unknown’ or ‘not occurred’ (as a result of simply being left un-ticked and translated into SDTM as a record showing that the event did not occur) and, thus, were not incorporated, as per OMOP CDM convention. The proportion of records that were not mapped as a result of being ‘unknown’ or ‘not done’ or having ‘not occurred’ is shown in Supplementary Tables 4–6. When these non-occurring events and event records with information that was irrelevant to analyses were excluded from calculations, 4% (OPUS), 2% (OrPHeUS) and 1% (EXPOSURE) of records were not mapped (Supplementary Tables 1–3).

## Discussion

In this study, novel approaches for converting SDTM data from three registry databases to the OMOP CDM were successfully developed and applied with limited deviation from the SDTM database content and very few records being excluded from mapping. This approach addresses a need for combining real-world data of patients with rare diseases for the purpose of evidence generation and could serve as reference for future researchers wishing to undertake similar data mapping projects.

Records from a total of 6457 patients in the OPUS (*n* = 2674), OrPHeUS (*n* = 3032), and EXPOSURE (*n* = 751) databases were successfully mapped. In total, 34.7% (OPUS), 67.4% (OrPHeUS) and 75.1% (EXPOSURE) of concept IDs for condition codes and 42.1% (EXPOSURE), 54.7% (OPUS) and 71.6% (OrPHeUS) of those for drug codes could be and were mapped to the OMOP CDM. There are several characteristics of registry data that explain these percentages. For example, the flexibility of SDTM clinical trial tabulation (e.g., free text and optional fields) means there are often missing or partially missing dates (obliging imputation rules), and there are often also multiple entries with the same meaning, which either have to be consolidated to the same concept ID or have to be differentiated through the use of FACT_RELATIONSHIP tables. Therefore, not all condition and drug codes could be mapped as unique entries with similar or identical meanings in the SDTM (for example, ‘unknown’, ‘unspecified’, ‘not available’ or ‘na’) mapped to the same concept_ID in the OMOP CDM. Consequently, there were fewer unique concept_IDs compared with unique source concept_IDs and this reflects that data are stored in a more consolidated form in the OMOP CDM, rather than data loss. Imputation rules and custom concepts were introduced to help achieve limited data exclusion. Imputation rules allowed for the retention of records with incomplete or missing dates, which are inherent to observational studies. Missing dates were especially prevalent in the OrPHeUS database wherein data were collected retrospectively from existing records in medical charts, which could be incomplete. In addition, data points occurring before study baseline often have partial or completely missing dates. All mapping and imputation rules were comprehensively documented, and were as consistent as possible across the registries. There will always be a certain level of imprecision associated with any imputation method but such imputations would be needed for analysis of the data regardless of whether data has been transformed to the CDM. Moreover, making imputation rules integral to the mapping process facilitates consistent analysis of the data.

Rare disease aetiologies are often not fully represented in medical vocabularies such as ICD and, as a result, custom concepts were required for this registry mapping process. In the OMOP CDM the standardized vocabulary is generally limited for rare diseases or rare subgroups of diseases, as the OMOP CDM was originally developed for electronic health records and claims data, which are based on ICD and other administrative codes [40, 41]. Standardized SNOMED concepts, like other administrative codes, are limited in their level of granularity and do not cover all rare diseases and disease subgroups. For instance, specific ICD and SNOMED terms do not exist for the different subgroups of PAH. To maintain PH disease classification and its clinically diverse subgroup information, custom concepts were introduced. An advantage of mapping multiple databases was that when a custom concept was introduced it could also be used for the mapping of subsequent databases. For example, the custom concept for drug- and toxin-induced PAH was first introduced in the OrPHeUS mapping, and was then also applied in the mapping of the OPUS and EXPOSURE databases. This process was already established by the HemOnc.org working group, which introduced vocabulary to standardize cancer conditions and treatments in the OMOP CDM that would allow structured analyses across registries [40, 41].

### Limitations

Limitations of the OMOP CDM format and the mapping process should be considered in advance of future work in this area, and the present study aims to give researchers a better understanding of said challenges and limitations. One limitation that researchers should consider is that several study groups may be mapping similar databases within the same disease area to OMOP CDM at the same time and using their own imputation rules and custom concepts. If such groups decide to collaborate, workshop discussions may be required to reach an agreement and develop guidelines on the methods used. To minimise this, we propose that aetiologies, treatments and procedures for rare diseases should be incorporated into the OMOP standardized vocabulary. In addition, we propose that the OMOP standardized vocabulary should be updated to capture procedures that have conclusively not been performed.

Currently, the OMOP CDM mapping convention is to only translate events that have actually occurred (or procedures that have actually been performed) and, as a result, there were substantial differences in the number of source records that could be mapped to the CDM. For example, in the prospective OPUS and EXPOSURE databases, study sites were able to confirm if a condition was present with a tick (or absence of a tick if not) that is stored as ‘yes’ or ‘no’ in SDTM format. As a result of ‘no’ answers not being mapped to the OMOP CDM, only 43.3% and 15.5% of unique source records for conditions were mapped for OPUS and EXPOSURE, respectively. In contrast, 99.2% of conditions were directly mapped to OMOP CDM for OrPHeUS, in which data were collected retrospectively and generally only conditions that had occurred were captured in the database. While these examples indicate consolidated data storage in the OMOP CDM,, it is well known that in clinical practice, assessments are often not systematically performed according to a strict protocol as in the case of randomized controlled trials [42] and it is sometimes advantageous to capture non-occurrence of events. Suggestions on how to retain this information (non-occurrence of events), when required, is available [43], but was not used in this work as recording the absence of events or conditions was not central to the aims of the original databases. It can provide valuable insights as, for example, the absence of an assessment could reflect real-world clinical practices or that the patient had severe disease, and this information will be mapped as part of our future work.

It is important to note that whilst we have performed quality checks on the mapped data, no research study has yet been performed on the mapped data in the OMOP CDM. Caution should be taken about the risk of oversimplifying interpretation of data and results after standardization. The mapping of OPUS, OrPHeUS, and EXPOSURE will allow us to perform many analyses in the common database structure. However, the choice of analysis should be carefully considered when dealing with multiple data sources in a CDM, as not all types of analysis can be feasibly or meaningfully applied to every data source or study design. It is crucial, therefore, to retain a certain level of nuance between databases that were originally designed for different purposes. For instance, disease-specific test results, such as the 6-min-walk distance (in metres) in PAH, should be analysed in disease-specific registries rather than in general administrative databases where such results are often not available or are incomplete. Similarly, claims and other administrative databases cover broader patient populations and, thus, are best positioned to compare a PH population with patients who have other types of respiratory disease or with ‘healthy’ patients.

### Outlook and future work

Our customized mapping methodology offers a foundation for future researchers wishing to map data from other registries or observational healthcare data sources. This foundation is of great value, particularly as no guidelines currently exist for the mapping of registry data to a CDM [32, 33]. One group has described the mapping of a single German PH registry to OMOP CDM but the details of the mapping process are largely limited to the ETL process [32]. Their process is ongoing and has involved adding 34 new concepts, 54 concept relationships, and 68 concept ancestor entries to the vocabularies, thereby achieving 100% coverage of the PH Nice classification in OMOP [32]. Continued sharing of methodologies and experiences could help avoid duplication of effort, shape future guidelines and foster collaborations. As such, we plan to collaborate with other research groups and external stakeholders that are interested in mapping registry data.

Within the OHDSI network, a working group is currently developing guidelines that may create a consensus on how to transform clinical trial data [44] (e.g., define observation period, store severity/seriousness, trial outcomes, planned information, dispensed but not administered medication data). These guidelines are an expansion beyond the previous norm of the OMOP being claims-focused and are an important first step for the handling of data from research studies, and will be a great help, though may not capture all challenges in mapping registry data. Other useful resources for researchers are systematic analysis tools such as ATLAS, an open-source software, which allows users to perform cross-database observational analyses to generate real-world evidence from patient-level data, without writing any programming code [45], and can then be applied in each database mapped to the OMOP CDM format. To summarise, researchers wishing to map registries should consider these guidelines, along with the experience described herein and by other study groups.

Importantly, our results show that the OMOP CDM can be used to store registry data without loss of essential information, and therefore add to previous reports of OMOP CDM use for coded, structured data (such as claims of electronic health records data), to demonstrate the value of OMOP CDM. For ongoing database studies, we would recommend refreshing the mapping as new data become available, considering potential changes in the data itself as well as changes in how the data have been captured. These changes may require additional custom mapping (for example, if a new medication or procedure is introduced), new imputation rules and customization of existing ETL logic and this could take time to discuss and implement. Mapping refreshes may also be performed when updates of the OMOP vocabularies are released. A study analysing the mapped data from the OPUS, OrPHeUS and EXPOSURE databases is needed and could be compared with existing, real-world, PH datasets such as those from the PHederation Network, for example [46]. In addition, our future work will include a study comparing SDTM- and OMOP CDM-based analysis results to further understand the variances in the datasets and confirm if results from the mapped registries are robust and suitable for review by payers and researchers.

## Conclusions

The success of our mapping process from the SDTM to the OMOP CDM in limiting exclusion of data has important implications for the generation of real-world evidence in both pulmonary hypertension and other rare diseases. Mapping registry data to the OMOP CDM facilitates more efficient collaborations between researchers and establishment of federated data networks, which is a major unmet need in rare diseases. Future researchers can apply our methods and solutions in mapping registry data in different disease areas with appropriate changes in customizations, as required by the nuances of the disease and/or study design.

## Supplementary Information

**Additional file 1.**

## Data Availability

The datasets generated and/or analysed during the current study are not publicly available due to only the mapping process of existing datasets being described but further information are available from the corresponding author on reasonable request.

## Abbreviations

CDM: Common data model
CRF: Case report form
CTEPH: Chronic thromboembolic pulmonary hypertension
EMA: European medicines agency
EnCePP: European network of centres for pharmacoepidemiology and pharmacovigilance
ETL,: Extract, transform, load
EU-ADR: European union adverse drug reactions
FDA: Food and drug administration
HCSRN: Health care systems research network
ICD: International classification of diseases
LOINC: Logical observation identifiers names and codes
MedDRA: Medical dictionary for regulatory activities
OHDSI: Observational health data sciences informatics
OMOP: Observational medical outcomes partnership
OPUS: Opsumit® users
OrPHeUS: Opsumit® historical users
PAH: pulmonary arterial hypertension
PCORnet: National patient-centred clinical research network
PH: Pulmonary hypertension
SDTM: Standards consortium study data tabulation model
SQL: Structured query language
SNOMED: Systematized nomenclature of medicine.
