# Supplementary material for: Standardizing registry data to the OMOP Common Data Model: experience from three pulmonary hypertension databases
Source: BMC Med Res Methodol. 2021 Nov 2;21:238. doi: 10.1186/s12874-021-01434-3 (PMC8565035; doi:10.1186/s12874-021-01434-3)
Supplement: Supplementary file 1 — Additional file 1. [file 12874_2021_1434_MOESM1_ESM.docx]

**Standardizing registry data to the OMOP Common Data Model: Experience from three pulmonary hypertension databases**

Patricia Biedermann^1^, Rose Ong^1^, Alexander Davydov^2^, Alexandra Orlova^2^, Philip Solovyev^2^, Hong Sun^1^, Graham Wetherill^1^, Monika Brand^1^, Eva-Maria Didden^1^

^1^Actelion Pharmaceuticals Ltd, Switzerland; ^2^Odysseus Data Services, Inc., MA, USA

**Supplementary materials**

**Supplementary Table 1.** Summary statistics for the main source tables that were mapped to OMOP *event* tables from the OPUS database

| **OPUS** | | | | | | |
| --- | --- | --- | --- | --- | --- | --- |
| **SDTM Source table** | **Source tables** | | | **OMOP CDM tables, n** | **Data excluded from source records to OMOP CDM tables, n** | **Actual data exclusion*, n** |
|  | **Source records, N** | **Events_lk records, n** | **Delta, n** |  |  |  |
| Adverse events | 48,360 | 48,360 | 0 | 46,536 | 1824 | 1824 |
| Concomitant medication | 23,656 | 23,656 | 0 | 20,068 | 3588 | 3588 |
| Death details | 1449 | 1449 | 0 | 1421 | 28 | 28 |
| Study medication exposure | 2729 | 2729 | 0 | 2720 | 9 | 9 |
| Laboratory data | 46,745 | 38,036 | 8,709 | 37,608 | 9137 | 428 |
| Medical History | 69,955 | 10,265 | 59,690 | 9637 | 60,318 | 628 |
| Vital Signs | 12,820 | 12,478 | 342 | 12,430 | 390 | 48 |
| PAH | 45,591 | 42,098 | 3,493 | 42,031 | 3560 | 67 |
| Substance use | 506 | 506 | 0 | 394 | 112 | 112 |
| Clinical events | 13,555 | 1590 | 11,965 | 1556 | 11,999 | 34 |
| Procedures | 6 | 6 | 0 | 6 | 0 | 0 |
| Findings about | 1387 | 335 | 1,052 | 265 | 1122 | 70 |
| Disposition | 5365 | 2681 | 2,684 | 2657 | 2708 | 24 |
| Demography | 2722 | 2684 | 38 | 2674 | 48 | 10 |
| **Total number of records** | **274,846** | **186,873** | **87,973** | **180,003** | **94,843** | **6870** |
| **Percentage difference** |  |  | **32%** |  | **35%** | **4%** |

***Data exclusion not including records that were excluded due to events considered as not having occurred.  *Source tables:* The following exclusions were applied for records i) with status ‘NOT DONE’ for laboratory, vital signs and PAH source tables; ii) with occurrence ‘N’ for the medical history source table.

*CDM Tables Final*: Duplicate records are excluded from OMOP CDM.
Clinical events are events collected post-baseline, typically comorbidities. ‘Findings about’ refers to Independent Liver Safety Data Review Board review of Hepatic Adverse Events of Special Interest, additional information about hepatic adverse events, and information about liver transplant (portopulmonary hypertension aetiology, liver transplant waiting list, Model for End-stage Liver Disease score).

OMOP CDM, Observational Medical Outcomes Partnership Common Data Model; OPUS, OPsumit USers; PAH, pulmonary arterial hypertension; SDTM, study data tabulation model.

**Supplementary Table 2.** Summary statistics for main source tables that were mapped to OMOP *event* tables from the OrPHeUS database

| **OrPHeUS** | | | | | | |
| --- | --- | --- | --- | --- | --- | --- |
| **SDTM Source table** | **Source tables** | | | **OMOP CDM tables, n** | **Data excluded from source records to OMOP CDM tables, n** | **Actual data exclusion *, n** |
|  | **Source records, N** | **Events_lk records, n** | **Delta, n** |  |  |  |
| Adverse events | 26,799 | 26,799 | 0 | 26,458 | 341 | 341 |
| Concomitant medication | 13,403 | 13,393 | 10 | 11,985 | 1418 | 1408 |
| Death details | 1473 | 1473 | 0 | 1438 | 35 | 35 |
| Study medication exposure | 3151 | 3151 | 0 | 3121 | 30 | 30 |
| Laboratory data | 48,260 | 48,260 | 0 | 47,350 | 910 | 910 |
| Medical History | 19,363 | 19,363 | 0 | 18,914 | 449 | 449 |
| Vital Signs | 112,573 | 104,599 | 7974 | 103,356 | 9217 | 1243 |
| PAH | 37,845 | 32,406 | 5439 | 32,126 | 5719 | 280 |
| **Total number of records** | **262,867** | **249,444** | **13,423** | **244,748** | **18,119** | **4696** |
| **Percentage difference** |  |  | **5%** |  | **7%** | **2%** |

***Data exclusion not including records that were excluded due to events considered as not having occurred.
Source tables: Difference between source values and Events_lk values as a result of records with status ‘NOT DONE’ have been excluded in the vital signs and PAH source tables, and duplicate records were filtered out from the concomitant medication table source if there were the same records in study medication exposure source table.

*OMOP CDM tables*: The following records were excluded i) those with duplicate patients, patients that do not meet inclusion and exclusion criteria, patients with missing macitentan initiation date; ii) those with missing start dates; iii) those with start and/or end dates prior to the recorded date of birth; iv) those with a start date after 31 December 2016; v) those with start / end date after the date of death; vi) those with the start date listed as after the end date; vii) duplicated records.

One source code may be mapped to more than 1 concept_ID, meaning that one source record may have several records in the OMOP CDM.

OMOP CDM, Observational Medical Outcomes Partnership Common Data Model; OrPHeUS, OPsumit Historical USers; PAH, pulmonary arterial hypertension; SDTM, study data tabulation model.

**Supplementary Table 3.** Summary statistics for main source tables that were mapped to OMOP *event* tables from the EXPOSURE database

| **EXPOSURE** | | | | | | |
| --- | --- | --- | --- | --- | --- | --- |
| **SDTM Source table** | **Source tables** | | | **OMOP CDM tables, n** | **Data excluded from source records to OMOP CDM tables, n** | **Actual data exclusion *, n** |
|  | **Source records, N** | **Events_lk records, n** | **Delta, n** |  |  |  |
| Adverse events | 1802 | 1802 | 0 | 1719 | 83 | 83 |
| Clinical events | 27,626 | 1815 | 25,811 | 1805 | 25,821 | 10 |
| Concomitant medication | 4668 | 4668 | 0 | 4596 | 72 | 72 |
| Death details | 51 | 51 | 0 | 51 | 0 | 0 |
| Disposition | 3024 | 3 | 3021 | 3 | 3021 | 0 |
| Study medication exposure | 1302 | 1115 | 187 | 1108 | 194 | 7 |
| Findings about events or interventions | 4844 | 4844 | 0 | 4823 | 21 | 21 |
| Functional tests | 2188 | 1240 | 948 | 1238 | 950 | 2 |
| Laboratory data | 2423 | 2423 | 0 | 2409 | 14 | 14 |
| Medical history | 755 | 755 | 0 | 751 | 4 | 4 |
| Procedures | 4547 | 823 | 3724 | 820 | 3727 | 3 |
| Respiratory measurements | 786 | 328 | 458 | 326 | 460 | 2 |
| Reproductive system findings | 1760 | 1456 | 304 | 1452 | 308 | 4 |
| Substance use | 2250 | 2010 | 240 | 1999 | 251 | 11 |
| Vital signs | 5825 | 5740 | 85 | 5716 | 109 | 24 |
| PAH therapy | 2899 | 1873 | 1026 | 1855 | 1044 | 18 |
| WHO functional class | 5551 | 4116 | 1435 | 3891 | 1660 | 225 |
| **Total number of records** | **72,301** | **35,062** | **37,239** | **34,562** | **37,739** | **500** |
| **Percentage difference** |  |  | **52%** |  | **52%** | **1%** |

***Data exclusion not including records that were excluded due to events considered as not having occurred.  *Source tables*: The following exclusions were applied for records i) with status ‘not done’ for the functional tests, respiratory measurements, vital signs and WHO functional class source tables; ii) with occurrence ‘N’, or ‘U’ for the procedures, substance use, clinical events and PAH therapy source tables; iii) with ‘%initiation at baseline’ category if there was a corresponding record with a ‘%treatment’ or ‘%therapy’ category for the study medication exposure and PAH therapy source tables; iv) with category ‘PAH-specific therapy intake during study’ for the PAH therapy source table; v) that are not new since last visit for the adverse events and clinical events source tables; vi) that have corresponding numbers of bleeding episodes in SUPPCE table for the clinical events source table; vii) with the test code ‘VSALL’ for the vital signs source table; viii) containing coronary or carotid revascularization procedures that are not new for the procedures source table; ix) with testcode ‘WORSEWHO’ for the WHO functional class source table; and x) with original results with status ‘NA’ for the reproductive system findings source table.

*OMOP CDM tables*: The following records were excluded i) those with patients that do not meet inclusion and exclusion criteria; ii) those with missing start dates; iii) those with start and/or end dates prior to the recorded date of birth; iv) those with a start date after study discontinuation; v) those with start / end date after the date of death; vi) those with the start date listed as after the end date; vii) duplicated records.

One source code may be mapped to more than 1 concept_ID, meaning that one source record may have several records in the OMOP CDM.

OMOP CDM, Observational Medical Outcomes Partnership Common Data Model; PAH, pulmonary arterial hypertension; SDTM, study data tabulation model; WHO, World Health Organization.

**Supplementary Table 4.** Proportion of tests that were 'not done' in OPUS source tables and contributed to the record exclusion shown in Supplementary Table 1

| **Source table** | **Number (%) of excluded records** | **Comment** |
| --- | --- | --- |
| Vital signs | 342 (2.7) | Test was not done |
| PAH (information on most recent RHC or pulmonary function test) | 3493 (7.7) | Test was not done |
| Laboratory data | 8709 (18.6) | Test was not done |
| Findings about | 376 (27.1) | Test was not done |
| Clinical events | 11,965 (88.0) | Event had never occurred |

PAH, pulmonary arterial hypertension; RHC, right heart catheterization

**Supplementary Table 5.** Proportion of tests that were 'not done' in OrPHeUS source tables and contributed to the record exclusion shown in Supplementary Table 2

| **Source table** | **Number (%) of excluded records** | **Comment** |
| --- | --- | --- |
| Vital Signs | 7974 (7.1) | Test was not done |
| PAH (information on most recent RHC or pulmonary function test) | 5439 (14.4) | Test was not done |

PAH, pulmonary arterial hypertension; RHC, right heart catheterization

**Supplementary Table 6.** Proportion of tests that were 'unknown' or 'not done' in EXPOSURE source tables and contributed to the record exclusion shown in Supplementary Table 3

| **Source table** | **Number (%) of excluded records** | **Comment** |
| --- | --- | --- |
| **Not done** | | |
| Functional Tests | 948 (43.3) | Test was not done |
| Respiratory Measurements | 458 (58.3) | Test was not done |
| Vital Signs | 85 (1.5) | Test was not done |
| WHO Functional Class | 683 (12.3) | Test was not done |
| Procedures | 3609 (79.4) | Event had never occurred |
| Clinical Events | 24645 (89.2) | Event had never occurred |
| PAH Therapy | 0 (0.0) | Event had never occurred |
| **Unknown** | | |
| Procedures | 17 (0.4) | Unknown if occurred |
| Substance Use | 240 (10.7) | Unknown if occurred |
| Clinical Events | 442 (1.6) | Unknown if occurred |

**Supplementary Table 7.** Ethics approval of the EXPOSURE study

| **Country** | **City/Site** | **Local Ethics Committee** |
| --- | --- | --- |
| Austria | Vienna | Ethics Committee of the Medical University of Vienna |
| Austria | Linz | Ethics Committee of the Medical University of Vienna |
| Austria | Innsbruck | Ethics Committee of the Medical University of Vienna |
| Austria | Graz | Ethics Committee of the Medical University of Vienna |
| Canada | Calgary | Conjoint Health Research Ethics Board (CHREB) |
| Canada | Ottawa | Ottawa Health Science Network Research Ethics Board (OHSNREB) |
| Canada | Hamilton | Hamilton Integrated Research Ethics Board (HiREB) |
| Canada | London | Western University office of Research Ethics |
| Canada | Quebec | Jewish General Hospital Research Ethics Board |
| Denmark | Aarhus | Data Protection Agency (Datatilsynet) |
| Finland | Turku | EETTINEN TOIMIKUNTA |
| Finland | Kuopio | EETTINEN TOIMIKUNTA |
| Germany | Kiel | Ethik-Kommission der Medizinischen Fakultät der Christian-Albrechts-Universität zu Kiel |
| Germany | Magdeburg | Ethik-Kommission der Otto-von-Guericke-Universität Magdeburg |
| Germany | Munich | Ethikkommission der Fakultät für Medizin der Technischen Universität München |
| Germany | Hannover | Ethik-Kommission der Medizinischen Hochschule Hannover |
| Germany | Solingen | Ethikkommission der Medizinischen Fakultät der Universität zu Köln |
| Germany | Leipzig | Ethik-Kommission an der Medizinischen Fakultät der Universität Leipzig |
| Germany | Lünen | Ethik-Kommission der Ärztekammer Westfalen-Lippe und der Westfälischen Wilhelms-Universität Münster |
| Germany | Dresden | Ethikkommission an der TU Dresden |
| Germany | Giessen | Ethik-Kommission des Fachbereichs Medizin der Justus-Liebig-Universität Gießen |
| Germany | Regensburg | Ethikkommission der Charité - Universitätsmedizin Berlin |
| Germany | Göttingen | Ethikkommission der Charité - Universitätsmedizin Berlin |
| Germany | Hamburg | Ethik-Kommission der Ärztekammer Hamburg |
| Germany | Homburg | Ethik-Kommission bei der Ärztekammer des Saarlandes |
| Germany | Greifswald | Ethik-Kommission an der Universitätsmedizin Greifswald Institut für Pharmakologie |
| Germany | Berlin | Ethikkommission der Charité - Universitätsmedizin Berlin |
| Germany | Mainz | Ethik-Kommission - Landesärztekammer Rheinland-Pfalz |
| Germany | Bonn | Ethikkommission an der Medizinischen Fakultät der Rheinischen Friedrich-Wilhelms-Universität Bonn |
| Germany | Berlin | Ethikkommission der Charité - Universitätsmedizin Berlin |
| Germany | Munich | Ethikkommission der Charité - Universitätsmedizin Berlin |
| Germany | Münster | Ethik-Kommission der Ärztekammer Westfalen-Lippe und der Westfälischen Wilhelms-Universität Münster |
| Germany | Aachen | Ethik-Kommission an der Medizinischen Fakultät der RWTH Aachen |
| Germany | Gerlingen | Ethik-Kommission bei der Landesärztekammer Baden-Württemberg |
| Germany | Koeln | Ethikkommission der Charité - Universitätsmedizin Berlin |
| Greece | Athens | Scientific Council (within the Hospital) |
| Greece | Ioannina | Scientific Council (within the Hospital) |
| Greece | Kallithea, Athens | Scientific Council (within the Hospital) |
| Greece | Thessaloniki, Greece | Scientific Council (within the Hospital) |
| Greece | Kallithea, Athens | Scientific Council (within the hospital) |
| Greece | Heraklion, Crete | Scientific Council (within the Hospital) |
| Greece | Thessaloniki, Greece | Scientific Council (within the Hospital) |
| Greece | Larissa | Scientific Council (within the Hospital) |
| Greece | Chaidari, Athens | Scientific Council (within the Hospital) |
| Italy | BOLOGNA | Comitato Etico AVEC c/o segreteria locale AOU di Bologna Policlinico S.Orsola-Malpighi |
| Italy | Nuoro | Comitato Etico ATS Sardegna |
| Italy | Bolzano | Comitato etico azienda sanitaria della provincia autonoma di Bolzano |
| Italy | Firenze | Comitato etico Area Vasta Centro-Segreteria del Comitato Etico, Pad. 3 - Nuovo Ingresso Careggi (NIC) - Didattica – |
| Italy | Milano | Comitato Etico IRCCS MulitMedica Sezione del Comitato Etico Centrale IRCCS Lombardia. |
| Italy | Milano | COMITATO ETICO IRCCS OSPEDALE SAN RAFFAELE |
| Italy | Brescia | Comitato Etico di Brescia |
| Italy | Genova | Comitato Etico Regionale IRCCS AOU S. Martino |
| Italy | Torrette-Ancona | Comitato Etico Regionale (C.E.R.) delle Marche |
| Italy | Fermo | Comitato Etico Regionale (C.E.R.) delle Marche |
| Italy | Catanzaro | Comitato Etico Regionale Calabria Area Centro |
| Italy | Vicenza | Comitato Etico per le sperimentazioni cliniche (CESC) della provincia di Vicenza |
| Italy | Milano | Comitato Etico Milano Area 2 |
| Italy | Bari | Comitato Etico AOU Consorziale Policlinico di Bari |
| Italy | Trieste | Comitato Etico Unico Regionale (C.E.U.R.) c/o Direzione Scientifica centro di riferimento oncologico Istituto di ricovero e cura a carattere scientifico |
| Italy | Bari | AOU Consorziale Policlinico di Bari |
| Italy | Udine | Comitato Etico Unico Regionale (C.E.U.R.) c/o Direzione Scientifica centro di riferimento oncologico Istituto di ricovero e cura a carattere scientifico |
| Italy | Milano | Comitato Etico Milano Area 2 |
| Italy | Acquaviva delle Fonti | Comitato Etico AOU Consorziale Policlinico di Bari |
| Italy | Novara | Comitato etico interaziendale presso AOU Maggiore della Carità di Novara |
| Italy | Verona | Comitato Etico per la Sperimentazione Clinica delle provincie di Verona e Rovigo |
| Italy | Macerata | Comitato Etico Regionale (C.E.R.) delle Marche |
| Italy | Padova | Comitato Etico per la Sperimentazione Clinica della provincia di Padova |
| Italy | Catania | Comitato Etico Catania 1 |
| Italy | Torino | Comitato Etico Interaziendale della Città della Salute e della Scienza di Torino |
| Netherlands | Nieuwegein | The University Medical Center Groningen(UMCG) |
| Netherlands | Maastricht | The University Medical Center Groningen(UMCG) |
| Netherlands | Amsterdam-Zuidoost | The University Medical Center Groningen(UMCG) |
| Slovakia | Bystrica | Eticka komisia/Ethics Committee Stredoslovensky ustav srdcovych a cievnych chorob, a.s. |
| Slovakia | Kosice | Etická komisia, Východoslovenský ústav srdcových a cievnych chorôb, a.s. |
| Slovakia | Bratislava | Etická komisia, Národný ústav srdcových a cievnych chorôb, a.s. |
| Slovakia | Bratislava | Etická komisia, Národný ústav srdcových a cievnych chorôb, a.s. |
| Spain | Madrid | CEIC Área 11 - Hospital 12 de Octubre |
| Spain | Madrid | CEIC Área 6 - Hospital Universitario Puerta de Hierro de Majadahonda |
| Spain | Pontevedra | Comité Autonómico de Ética de la Investigación de Galicia |
| Spain | Madrid | CEIC Area 1 - Hospital General Universitario Gregorio Maranon |
| Spain | Valencia | CEIC Hospital Universitari i Politècnic La Fe |
| Spain | Santander | Comité Etico de Investigacion Clínica de Cantabria |
| Spain | Santiago | Comité Autonómico de Ética de la Investigación de Galicia |
| Spain | Madrid | CEIC Área 4 - Hospital Universitario Ramón y Cajal |
| Spain | Valencia | CEIC Hospital Universitari i Politècnic La Fe |
| Spain | Barcelona | CEIC Hospital Universitari de Bellvitge |
| Spain | Alcoy | CEIC HOSPITAL DE ALCOY |
| Spain | Palma de Mallorca | Comité de Ética de la Investigación de las Illes Balears |
| Spain | Málaga | CEI Provincial de Málaga |
| Spain | Málaga | CEI Provincial de Málaga |
| Spain | Barcelona | CEIC Hospital Universitari Vall d Hebron |
| Spain | Salamanca | CEIC Area de Salud de Salamanca |
| Spain | Barcelona | CEIC Hospital Universitari Vall d Hebron |
| Spain | Zaragoza | Comité Etico de Investigacion Clínica de Aragon - CEICA |
| Spain | Oviedo | Comité Ético de Investigación Clínica de Asturias |
| Spain | Sevilla | CEIm de los Hospitales Universitarios Virgen Macarena-Virgen del Rocío |
| Spain | Valladolid | CEIm Área de Salud Valladolid Este |
| Spain | Las Palmas | CEIm Provincial de Las Palmas |
| Spain | Barcelona | CEIC de la Corporacio Sanitaria de Parc Taulí |
| Spain | Barcelona | CEIC Hospital Clinic de Barcelona |
| Spain | Zaragoza | Comité Ético de Investigación Clínica de Aragón (CEICA) |
| Spain | Barcelona | Comité de Ética de la Investigación del Hospital U. Germans Trias i Pujol |
| Spain | Barcelona | CEIm del Institut Hospital del Mar d'Investigacions Mèdiques |
| Sweden | Göteborg | Etikprövningsmyndigheten |
| Sweden | Umeå | Etikprövningsmyndigheten |
| Sweden | Uppsala | Etikprövningsmyndigheten |
| Switzerland | Zürich | Kanton Zurich, Kantonale Ethikkommission |

**Supplementary Appendix**

*Mapping Methods*

For the mapping described herein, OMOP CDM version 5.3.1 with recent OMOP vocabulary used (systematized nomenclature of medicine [SNOMED] International edition, July 2019 release for EXPOSURE and January 2020 release for OPUS and OrPHeUS; Medical Dictionary for Regulatory Activities [MedDRA™] version 22.0 for EXPOSURE and version 23.0 for OPUS and OrPHeUS; RxNorm September 2019 release for EXPOSURE and 5 April 2020 release for OPUS and OrPHeUS; and Logical Observation Identifiers Names and Codes [LOINC] version 2.67). The extract, transform, load (ETL) process was performed on the Amazon Elastic MapReduce cluster through conversion scripts written in Spark Structured Query Language (SQL) with a custom-made automatization utility called CDM Kit, which was written in Python™ with the use of Apache Spark™ application programming interface.

An overview of the 7-phase process of mapping registry data in the SDTM format to the OMOP CDM is shown in **Figure 2**. In the first, pre-analysis phase, source documentation and the SDTM were reviewed and a list of questions to discuss with the source data experts was prepared. From this, initial matching of source tables to OMOP CDM tables was performed and a list of custom vocabularies and sets to values to be custom mapped by medical experts was determined. The custom mapping of source values was performed in Phase 2 of the process. The ETL specification was devised in Phase 3 and questions about source data such as imputation rules, duplications in source tables and observation period logic were discussed with source data experts. Phase 4 represented the development process, in which SQL scripts were written to convert source tables into the OMOP CDM based on ETL specification, OMOP CDM standardized vocabularies were uploaded, and custom mapping was provided by medical experts. A specific example of mapping source tables to OMOP CDM tables is shown in **Figure 3**. Quality assessments and control were performed in Phase 5, including unit tests (to check OMOP CDM requirements were met), integration tests (project-specific tests to check that SQL scripts met requirements in the ETL specification. Descriptive statistics for the source data and OMOP CDM tables were also obtained in Phase 5. OMOP CDM was released in Phase 6, including release notes, OMOP CDM tables, ETL specification and html reports. The seventh and final phase was user acceptance testing: end users tested the OMOP CDM tables by comparing results of analyses based on source data and on OMOP CDM tables. In addition, any bugs were fixed and change requests identified in Phases 3 to 6 were implemented, and final documentation was prepared.

Currently, all-level MedDRA terms, which are used in the OPUS, OrPHeUS and EXPOSURE databases, are not an OHDSI-supported vocabulary but are considered ‘classification concepts’ in OMOP vocabularies. Classification concepts are non-standard and do not represent the data in the OMOP CDM, but instead are part of the standard concepts hierarchy, and can be used for hierarchical queries to search for a certain concept (e.g. for a specific medical condition, such as idiopathic PAH). However, classification concepts technically can be used as source concepts to populate the source concept identification (source_concept_ID) field of the event tables [1]. Thus, both standard equivalent concepts (with the SNOMED [2] hierarchy) and source MedDRA concepts (with a different MedDRA hierarchy) can be used for analysis [1]. However, as classification concepts, MedDRA terms have no full equivalent ‘maps to’ links to standard concepts, meaning that the MedDRA codes have no direct translation to OMOP standardized vocabulary for conditions and laboratory data (SNOMED, LOINC) and, thus, a degree of manual mapping was required. This process is described in further detail in the results.

**References**

1. Observational Health Data Sciences and Informatics, The Book of OHDSI, 2020. Available at: <https://ohdsi.github.io/TheBookOfOhdsi/> [Last accessed November 2020]. In*.*

2. Systematised nomenclature of medicine [<http://www.snomed.org/>]
